# Robotic Surgery Improves Technical Performance and Enhances Prefrontal Activation During High Temporal Demand

**DOI:** 10.1007/s10439-018-2049-z

**Published:** 2018-06-04

**Authors:** Harsimrat Singh, Hemel N. Modi, Samriddha Ranjan, James W. R. Dilley, Dimitrios Airantzis, Guang-Zhong Yang, Ara Darzi, Daniel R. Leff

**Affiliations:** 1 Hamlyn Centre for Robotic Surgery, Imperial College London, London, UK; 2 Department of Surgery and Cancer, Imperial College London, London, UK; 3 Institute for Liver and Digestive Health (ILDH), University College London, London, UK; 4 Department of Surgery and Cancer, St Mary’s Hospital, 2nd Floor, Paterson Wing, Praed Street, London, W2 1NY UK

**Keywords:** Neuroimaging, Brain function, Stress, Cognitive workload, Surgical skills, Laparoscopy, Suturing

## Abstract

Robotic surgery may improve technical performance and reduce mental demands compared to laparoscopic surgery. However, no studies have directly compared the impact of robotic and laparoscopic techniques on surgeons’ brain function. This study aimed to assess the effect of the operative platform (robotic surgery or conventional laparoscopy) on prefrontal cortical activation during a suturing task performed under temporal demand. Eight surgeons (mean age ± SD = 34.5 ± 2.9 years, male:female ratio = 7:1) performed an intracorporeal suturing task in a self-paced manner and under a 2 min time restriction using conventional laparoscopic and robotic techniques. Prefrontal activation was assessed using near-infrared spectroscopy, subjective workload was captured using SURG-TLX questionnaires, and a continuous heart rate monitor measured systemic stress responses. Task progression scores (au), error scores (au), leak volumes (mL) and knot tensile strengths (N) provided objective assessment of technical performance. Under time pressure, robotic suturing led to improved technical performance (median task progression score: laparoscopic suturing = 4.5 vs. robotic suturing = 5.0; *z* = − 2.107, p = 0.035; median error score: laparoscopic suturing = 3.0 mm vs. robotic suturing = 2.1 mm; *z* = − 2.488, *p* = 0.013). Compared to laparoscopic suturing, greater prefrontal activation was identified in seven channels located primarily in lateral prefrontal regions. These results suggest that robotic surgery improves performance during high workload conditions and is associated with enhanced activation in regions of attention, concentration and task engagement.

**Electronic supplementary material:**

The online version of this article (10.1007/s10439-018-2049-z) contains supplementary material, which is available to authorized users.

## Introduction

**Figure 1:**
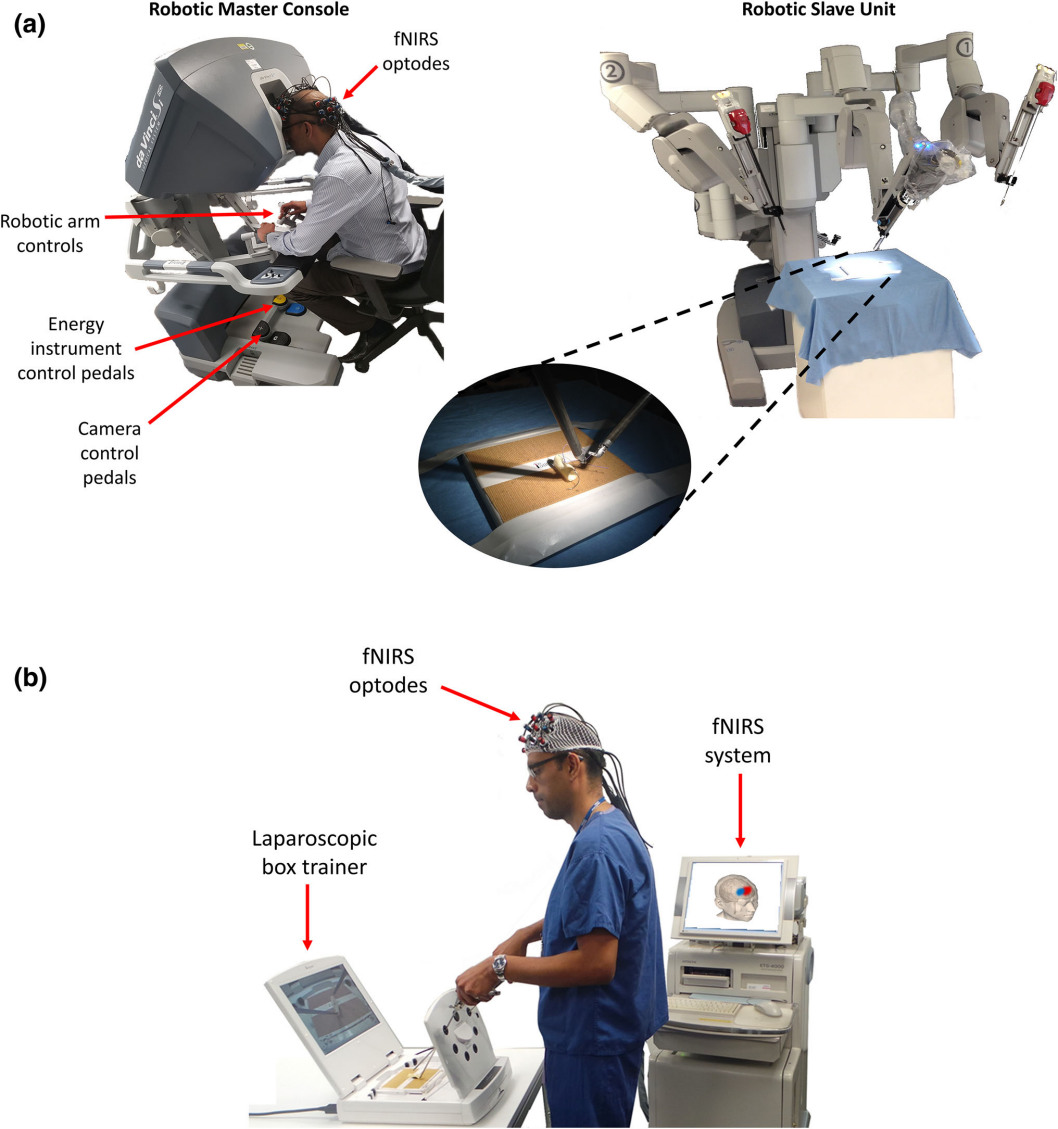

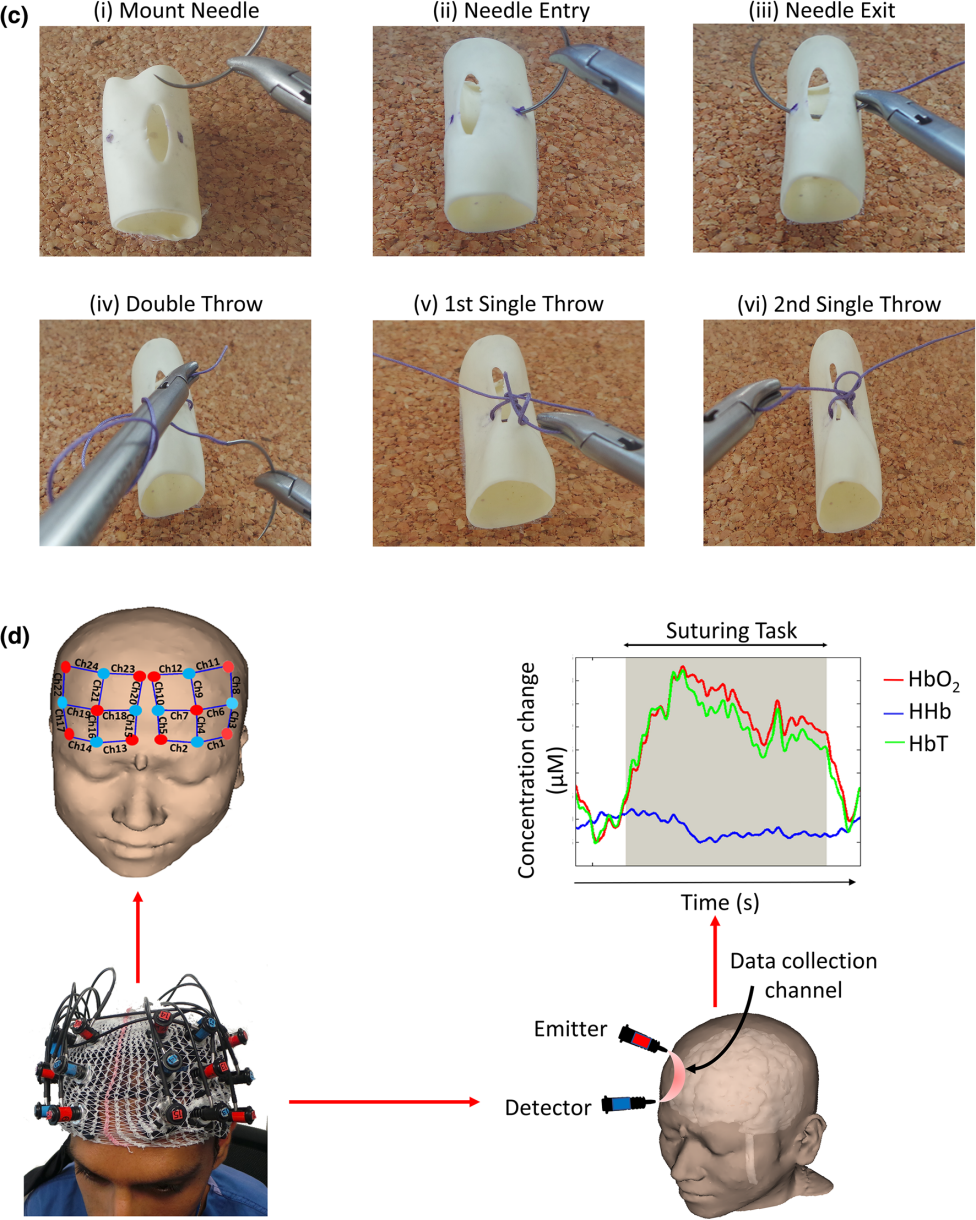
(**a**) The da Vinci^®^ Si system (Intuitive Surgical Inc, Sunnyvale, CA) consists of a master console system with which the surgeon controls a slave unit comprising robotic arms that move around fixed pivot points and carry out the surgeon’s commands; (**b**) A bench-top box trainer (iSim2, iSurgicals, UK) used for the laparoscopic suturing task; (**c**) Key steps of the suturing task in which a reef knot is created: (i) mounting the needle onto the needle holder, (ii) inserting the needle into the Penrose drain as close to pre-marked target points as possible, (iii) exiting the needle out of the drain as close to pre-marked target points as possible, (iv) double throw of suture thread, (v) first single throw, and (vi) second single throw; (**d**) Prefrontal activation during the task was assessed using functional near-infrared spectroscopy (fNIRS), a non-invasive neuroimaging technique, which measures differences between emitted and detected near infrared light to estimate the local concentration changes of oxygenated haemoglobin (HbO_2_) and deoxygenated haemoglobin (HHb), as a surrogate of brain activation. The 3D head reconstruction demonstrates the 3 × 3 arrays of optodes located over the left and right PFC, along with the positions of 24 corresponding channels (‘Ch’) that measure haemodynamic responses in an area of cortex located between an emitter (red) and detector (blue). The typical cortical haemodynamic response in channels exhibiting activation comprises an increase in HbO_2_, a smaller decrease in HHb, and an increase in total haemoglobin (HbT = HbO_2_ + HHb).

The propagation of robotic techniques has revolutionised minimal access surgery by addressing some of the limitations of the laparoscopic approach.^20^ Robotic technologies do not replace the surgeon nor perform tasks independently,^42^ but rather provide complementary capabilities that enhance dexterity and improve ergonomic efficiency.^40^ Since they are controlled by the surgeon, they are often described as ‘*master*–*slave systems’* and composed of two components: (1) the *master console* which is the user interface that provides the surgeon with a 3-dimensional view of the operating field, manipulators which allow the surgeon to remotely control instruments, and a control panel allowing adjustment of camera focus and position; and (2) the *slave unit* positioned at the patient’s side on which the camera and instruments are docked and manipulated on robotic arms (Fig. 1a).

Robotic-assisted surgery has been increasingly employed in a number of complex procedures, particularly those in which the operative field is confined such as colorectal, gynaecological, and urological surgeries.^20^ The growth of robotic platforms in surgery is supported by a wealth of literature demonstrating its advantages over conventional laparoscopy.^20^ Robotic instruments have six degrees of freedom, greater than conventional laparoscopic instruments, which improves instrument manipulation. Software filters minimize hand tremors normally amplified in conventional laparoscopy. Finally, motion scaling allows large hand movements at the master console to be translated to micromovements of the instruments within the patient. A 3-dimensional field of view improves depth perception which, coupled with an absence of a fulcrum effect, makes instrument movement more intuitive and improves hand–eye coordination. Robotic platforms are ergonomically efficient and reduce physical burden on the surgeon as the operator is seated comfortably at a remote master console in contrast to the awkward positions frequently required during laparoscopic surgery.^40^ In contrast to laparoscopy, camera motion in robotic surgery is steady and controlled by the primary surgeon *via* the master console system. Furthermore, simulation studies demonstrate the robotic approach improves smoothness of movement,^10^ reduces instrument pathlength^10^ and errors of commission,^31^,^37^ and leads to learning curve attenuation compared to laparoscopy.^10^,^37^

Several studies have investigated surgeons’ mental demands and have demonstrated robotic surgery to be less cognitively demanding and/or stressful than laparoscopy based on both subjective questionnaires^31^,^37^,^40^ as well as cardiovascular parameters.^31^,^40^ For example, Moore *et al*.,^31^ utilised the Surgical Task Load Index, the Rating Scale for Mental Effort, and assessment of heart rate variability to demonstrate robotic-assisted surgery to be less stressful than the laparoscopy during ball pick-and-drop and rope threading tasks.^31^ Similarly, Hubert *et al*.,^14^ observed that physical workload (electromyography), stress (heart rate) and perceived mental effort (NASA Task Load Index) were greater during laparoscopy compared to robotic-assisted tasks.^14^ Since intraoperative stress and high cognitive workload can be detrimental to surgical performance,^4^ the suggestion that robotic platforms offload mental demands compared to laparoscopy is appealing. However, to date, no studies have directly objectively assessed the impact of robotic technology on surgeons’ brain function as means of confirming or refuting this hypothesis.

Neuroimaging has been used to assess brain function during mentally demanding tasks, such as working memory tasks^5^,^6^ and simulated command and control tasks.^6^,^16^ The majority of this literature places emphasis on the prefrontal cortex (PFC)—an area of the brain important for executive control, attention and task engagement^28^—and suggests an inverted-U shaped relationship between PFC activation and mental workload, whereby activation initially increases with workload,^6^ but then diminishes at excessive levels of mental demand.^13^,^16^ The use of brain imaging techniques to investigate surgeons’ cognition is rapidly expanding^30^ and has deepened our understanding of technical skill acquisition,^22^ decision-making processes^24^ and the effects of fatigue.^23^ Although there is limited evidence on the impact of intraoperative mental demands on operator brain function, our previous work demonstrated that intraoperative temporal stress prompts attenuated prefrontal activations and technical performance deterioration during a laparoscopic suturing task.^29^ However, there are no studies reporting prefrontal responses during robotic surgery hence the impact of robotics on prefrontal function under mental demands remains unknown.

Several studies from outside medicine have shown that more mentally demanding conditions are associated with less prefrontal activation and a decline in task performance.^1^,^13^,^16^,^26^,^34^ In particular, several fNIRS studies demonstrate tasks performed under stress or heightened cognitive load impact adversely on prefrontal responses, including n-back working memory tasks,^26^ mental arithmetic tasks performed under time pressure and with negative feedback,^1^ multitasking during naval air warfare management,^16^ and simulated piloting tasks under variable demand.^13^ Together, these studies suggest that conditions in which mental demands are greatest lead to reduced attention, manifest as diminished prefrontal engagement, and a deterioration in task performance. Conversely, less cognitively demanding conditions are associated with greater prefrontal activation and improved task engagement. Given that as discussed, robotic platforms are found to offload mental demands versus laparoscopy^14^,^31^,^37^,^40^ and highly stressful or demanding operative conditions are associated with attenuated prefrontal responses,^1^,^13^,^16^,^26^,^34^ we hypothesise that, compared to conventional laparoscopy, robotic surgery will result in greater prefrontal activation indicative of greater task engagement and attentional control, and improved technical performance when suturing under time pressure. The prediction being that improved instrument ergonomics and alleviation of workload is manifest as improved technical performance and enhanced activation within executive control centres. Therefore, the aim of the current study is to compare the impact of robotic surgery and conventional laparoscopy on prefrontal activation in surgeons performing an intracorporeal suturing task under temporal demand.

## Materials and Methods

### Subjects

Following local ethical approval (LREC: 05/Q0403/142), 102 surgeons from across London were invited to enrol in this study. Subjects were only eligible to participate if they were higher surgical trainees or consultants. Eight surgeons (1 consultant, 7 trainees) agreed to take part (mean age ± SD = 34.5 ± 2.9 years, male:female ratio = 7:1) and gave written informed consent prior to the study commencing. Subjects had significantly greater previous experience with laparoscopic suturing compared with robotic suturing [median number of times previously performed (IQR): laparoscopic suturing = 12.5 (10.0–22.5), robotic suturing = 1.5 (0.0–3.8); *p* = 0.012]. Participants were screened for handedness [median handedness (range) = 0.80 (− 0.20 to 1.00)] and neuropsychiatric illness (*n* = 0), and were asked to refrain from consuming alcohol or caffeine for the preceding 24 h.

### Suturing Task and Experimental Paradigm

The experiment was conducted in a block design in which each participant performed the surgical task five times in each condition (order randomized) on both surgical platforms with an inter-knot rest period of 30 s. The surgical task involved intracorporeal suturing with a 2-0 Vicryl^®^ suture (Ethicon, Somerville, NJ) inserted as close as possible to pre-marked entry and exit points on either side of a defect in a Penrose drain. Tying a knot required formulation of one double throw followed by two single throws of the suture. Each subject executed the experimental paradigm first using a conventional laparoscopic approach on a box trainer (iSim2, iSurgicals, UK) and, after a washout period of 6 months, repeated the experiment *via* robotically-assisted technique using a da Vinci^®^ Si system (Intuitive Surgical Inc, Sunnyvale, CA). All participants performed the task under two experimental conditions on each platform, as follows: (1) *‘self*-*paced’* in which subjects took as much time as they needed to tie a knot, and (2) *‘time pressure’* where a time limit of 2 min per knot was imposed (Fig. 1). During the self-paced condition, the task episode was terminated if a subject required longer than 5 min to complete the knot. During the inter-trial rest periods, subjects were instructed to assume a comfortable seated position looking directly ahead at a blank screen and avoid thoughts relating to the task.

### Neuroimaging Data

Functional near infrared spectroscopy (fNIRS) is a non-invasive functional neuroimaging technique which measures cortical absorption of near infrared (NIR) light to estimate the local concentration changes of oxygenated haemoglobin (HbO_2_) and deoxygenated haemoglobin (HHb). The typical haemodynamic brain activation response comprises a task-evoked increase in HbO_2_ and a lower amplitude decrease in HHb. An ETG-4000 Optical Topography System (Hitachi Medical Co, Japan) was used to simultaneously measure both HbO_2_ and HHb changes across 24 prefrontal cortical locations (*‘channels’*), the positions of which were defined according to the international 10–20 system of probe placement.^18^

### Stress and Technical Performance

The Surgical Task Load Index (SURG-TLX) questionnaire^41^ was used to evaluate subjective workload. Heart rate (HR) was continuously recorded using a wireless monitor (Bioharness, Zephyr Technology, USA) to provide a physiological measure of stress. Technical skill was objectively assessed using four performance parameters described in our previous work:^29^

#### Task Progression Score (TPS; Arbitrary Units, au)

Each task episode was assigned a score based on task progression, with one point awarded for each of the following steps: (1) mounting the needle onto the needle holder, (2) needle insertion into the drain, (3) exiting the needle from the drain, (4) double throw, (5) 1st single throw, and (6) 2nd single throw of a laparoscopic reef knot. The TPS comprised the total number of points obtained during the task (maximum score = 6).

#### Error Score (ES; mm)

Adapted from the FLS scoring system for intracorporeal suturing, the ES was calculated as follows: Error Score = [distance (mm) between needle insertion point and pre-marked target position + distance (mm) between needle exit point and pre-marked target position]. Accurate needle placement *in vivo* is necessary as else there is risk of damage to surrounding structures.

#### Leak Volume (LV; mL)

Saline was infused through each drain at a rate of 150 drops/min controlled *via* a digital pump. The volume of saline leaking from the closed defect over a 1 min period was recorded to assess the quality of defect closure. Lower leak volumes would reflect superior defect closure, analogous to improved ligation security of a bleeding vessel.

#### Knot Tensile Strength (KTS; Newtons, N)

A bench-top tensiometer (5565 single-axis tensiometer, Instron, UK) was used to quantify the tensile strength of each tied knot. Greater knot tension favours knot security, an important aspect of sound surgical technique.

### Data Processing and Statistical Analysis

Statistical analysis was performed using SPSS version 23.0 (IBM Corp., Armonk, NY). A threshold *p* < 0.05 was deemed statistically significant.

#### Subjective Workload and Technical Skills Data

Between-condition and within-condition comparisons were performed using the paired samples *t* test for parametric data (*i.e.,* SURG-TLX and leak volume) and the Wilcoxon Signed Ranks test for non-parametric data (*i.e.,* heart rate, task progression score, error score and knot tensile strength).

#### Neuroimaging Data

Data were processed using HOMER2, an open source MATLAB-based toolbox.^15^ High-frequency noise and electrocardiographic effects were minimized using a low-pass filter (0.5 Hz). Channel rejection was based on amplitude thresholding and a signal-to-noise ratio of > 2. Furthermore, motion artefacts were detected and corrected using spline interpolation of optical density data. The modified Beer-Lambert law was used to convert changes in light intensity into HbO_2_ and HHb concentration changes.^11^ Data were then processed for statistical analysis using a bespoke analytical framework (ICNNA)^32^ and self-paced blocks were resampled to 120 s, ensuring uniformity with time pressure blocks, before collating a database of per subject HbO_2_ and HHb values for each condition, operative platform and block. A time window of 120 s with a break delay of 10 s from task onset was selected for this purpose.

#### Identification of Channel Activation

For each operative platform (laparoscopic and robotic) and each condition (SP and TP), channel activation was determined by comparing the mean baseline rest Hb data sampled over 10 s before task onset (HbO_2Rest_ and HHb_Rest_) with mean task Hb data sampled over 110 s starting 10 s after task onset (HbO_2Task_ and HHb_Task_) using the Wilcoxon Signed Ranks test. Channels displaying a statistically significant (*p* < 0.05) increase in HbO_2_ and decrease in HHb were considered “activated”, and those showing a significant decrease in HbO_2_ and increase in HHb were considered “deactivated”.

#### Comparison of Activation Responses

For each channel, new variables ΔHbO_2_ (HbO_2Task_ − HbO_2Rest_) and ΔHHb (HHb_Task_ − HHb_Rest_) were computed. Using the Wilcoxon Signed Ranks test, ΔHbO_2_ and ΔHHb in each channel were compared between conditions (*i.e.,* SP vs. TP) for each platform; and between platforms (*i.e.,* laparoscopic vs. robotic) for each experimental condition.

#### Correlation Analysis

In order to assess the extent of systemic contribution to the cortical haemodynamic signal, channel-wise correlations between heart rate and changes in oxygenated haemoglobin were performed on a subject-level for each platform and each condition.

## Results

### Self-Paced vs. Time Pressure (Laparoscopic Suturing)

#### Subjective Workload and Heart Rate

**Figure 2:**
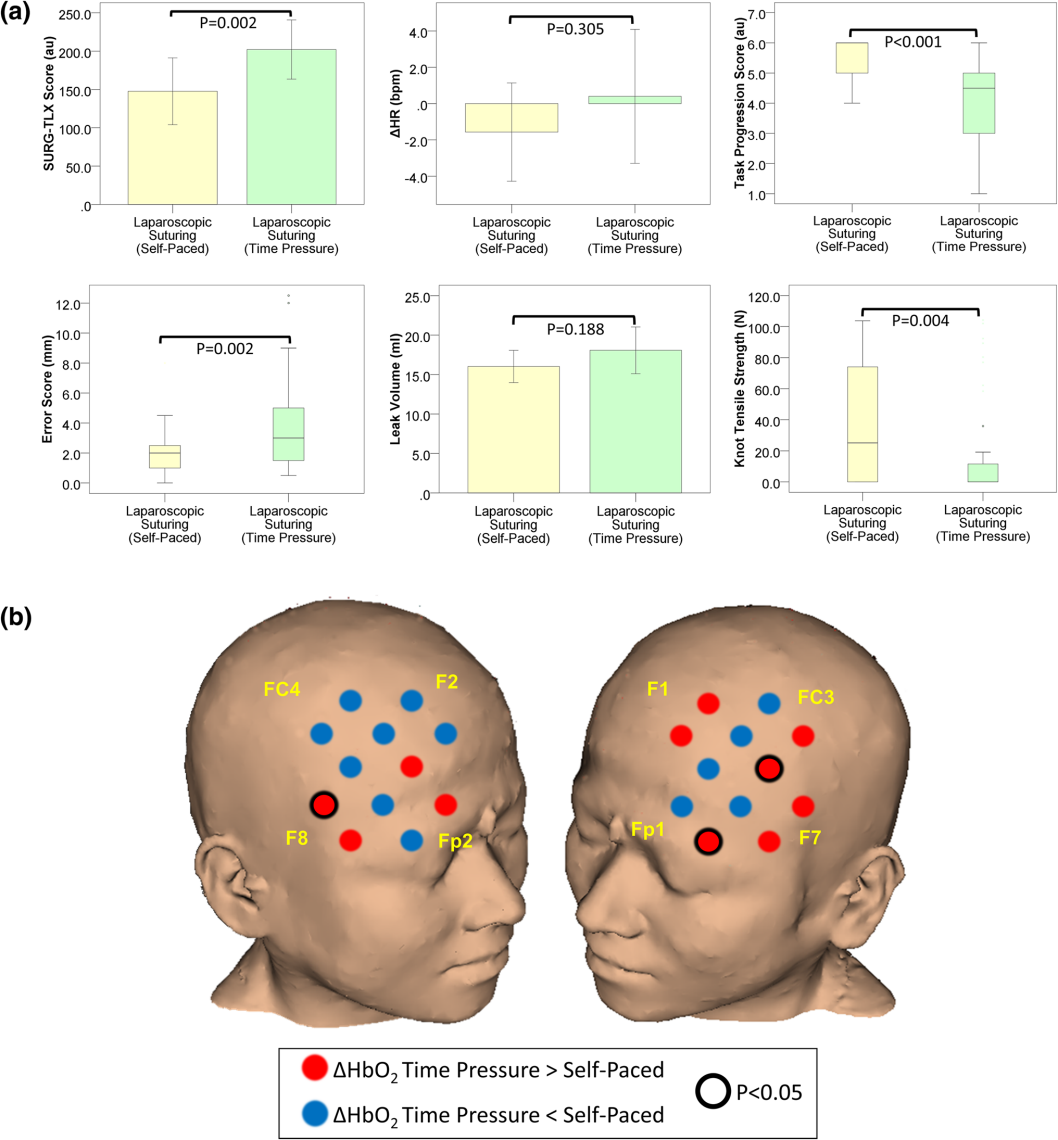
(**a**) Subjective workload (SURG-TLX) scores, heart rate, and technical performance in self-paced (yellow bars) and time pressure (green bars) conditions during laparoscopic suturing. Error bars represent the 95% confidence interval; (**b**) Comparison of the HbO_2_ response (ΔHbO_2_) in time pressure and self-paced conditions during laparoscopic suturing. Channels exhibiting a smaller HbO_2_ response in the time pressure compared to the self-paced condition are blue, whereas those exhibiting a smaller response in the self-paced condition are red. Channels in which there was a significant difference (*p* < 0.05, Wilcoxon Signed Ranks test) in the HbO_2_ response between conditions are outlined (black circle). Reference points of the 10–20 system of optode placement are shown in yellow (right PFC: Fp2 = source 6, F8 = source 7, F2 = source 9, FC4 = source 10; left PFC: Fp1 = source 2, F7 = source 1, F1 = source 5, FC3 = source 4). *Au* arbitrary units, *ΔHR* change in heart rate from rest to task, *bpm* beats per minute, *mm* millimetres, *mL* millilitres, *N* Newtons.

Subjective workload was significantly greater in the TP compared to SP condition (mean SURG-TLX ± SD: SP = 147.6 ± 52.2, TP = 202.1 ± 46.2; *t*(7) = − 4.805, *p* = 0.002). However, as illustrated in Fig. 2a and summarised in Table 1, there was no significant difference in ΔHR between conditions (*p* = 0.305).

**Table 1:** Workload, heart rate and performance during laparoscopic and robotic suturing in self-paced and time pressure conditions.

	Self-paced						Time pressure					
	SURG-TLX score (au)	ΔHR (bpm)	Task progression score (au)	Error score (mm)	Leak volume (mL)	Knot tensile strength (N)	SURG-TLX Score (au)	ΔHR	Task progression score (au)	Error score (mm)	Leak volume (mL)	Knot tensile strength (N)
Laparoscopic suturing	147.6 (52.2)	− 1.6 (3.2)	6.0 (1.0)	2.0 (2.0)	16.0 (2.4)	25.1 (74.4)	202.1 (46.2)	0.4 (4.4)	4.5 (2.0)	3.0 (3.6)	18.1 (3.5)	0.0 (15.4)
Robotic suturing	115.9 (72.1)	1.7 (2.5)	6.0 (0.0)	1.9 (1.8)	15.5 (1.3)	21.2 (52.8)	183.9 (75.9)	1.0 (1.5)	5.0 (2.0)	2.1 (1.9)	18.3 (1.2)	1.4 (13.1)

*SURG-TLX* Surgical Task Load Index, *ΔHR* change in heart rate from rest to task, *au* arbitrary units, *bpm* beats per minute, *mm* millimetres, *mL* millilitres, *N* Newtons. SURG-TLX, ΔHR and Leak Volume data are mean values (SD). Task Progression Score, Error Score and Knot Tensile Strength data are median values (IQR)

#### Technical Performance

Performance was significantly inferior in the TP compared to the SP condition [task progression score (median (IQR): SP = 6.0 (1.0) vs. TP = 4.5 (2.0); z = − 4.710, *p* < 0.001), error score (median (IQR): SP = 2.0 (2.0) mm vs.TP = 3.0 (3.6) mm; *z* = − 3.084, *p* = 0.002), and knot tensile strength (median (IQR): SP = 25.1 (74.4) N vs. TP = 0.0 (15.4) N; *z* = − 2.843, *p* = 0.004)]. As illustrated in Fig. 2a and summarised in Table 1, there was no significant between-condition difference in leak volume (*p* = 0.188).

#### Cortical Haemodynamic Response

The magnitude of channel activation varied depending on task demand, with a greater proportion of channel activation observed in SP versus TP. In the SP condition, twenty out of twenty-four channels exhibited a task-induced increase in HbO_2_ concentration, two of which reached significance (channels 20 and 21). Similarly, twenty channels exhibited a decrease in HHb concentration, of which eleven reached statistical significance. Two channels (channels 20 and 21) demonstrated both a significant increase in HbO_2_ as well as a significant decrease in HHb. Task-induced increase in HbO_2_ was observed in twenty channels in the TP condition, one of which reached significance (channel 6). Fifteen channels exhibited HHb decreases, six of which were statistically significant. No channels demonstrated a simultaneous increase in HbO_2_ and decrease in HHb. Between-condition comparisons of ΔHbO_2_ revealed attenuated responses in TP compared to SP in thirteen channels, located predominately in the bilateral VLPFC and DLPFC (Fig. 2b). Analysis of ΔHHb responses demonstrated diminished responses in TP compared to SP in seventeen channels.

### Self-Paced vs. Time Pressure (Robotic Suturing)

#### Subjective Workload and Heart Rate

**Figure 3:**
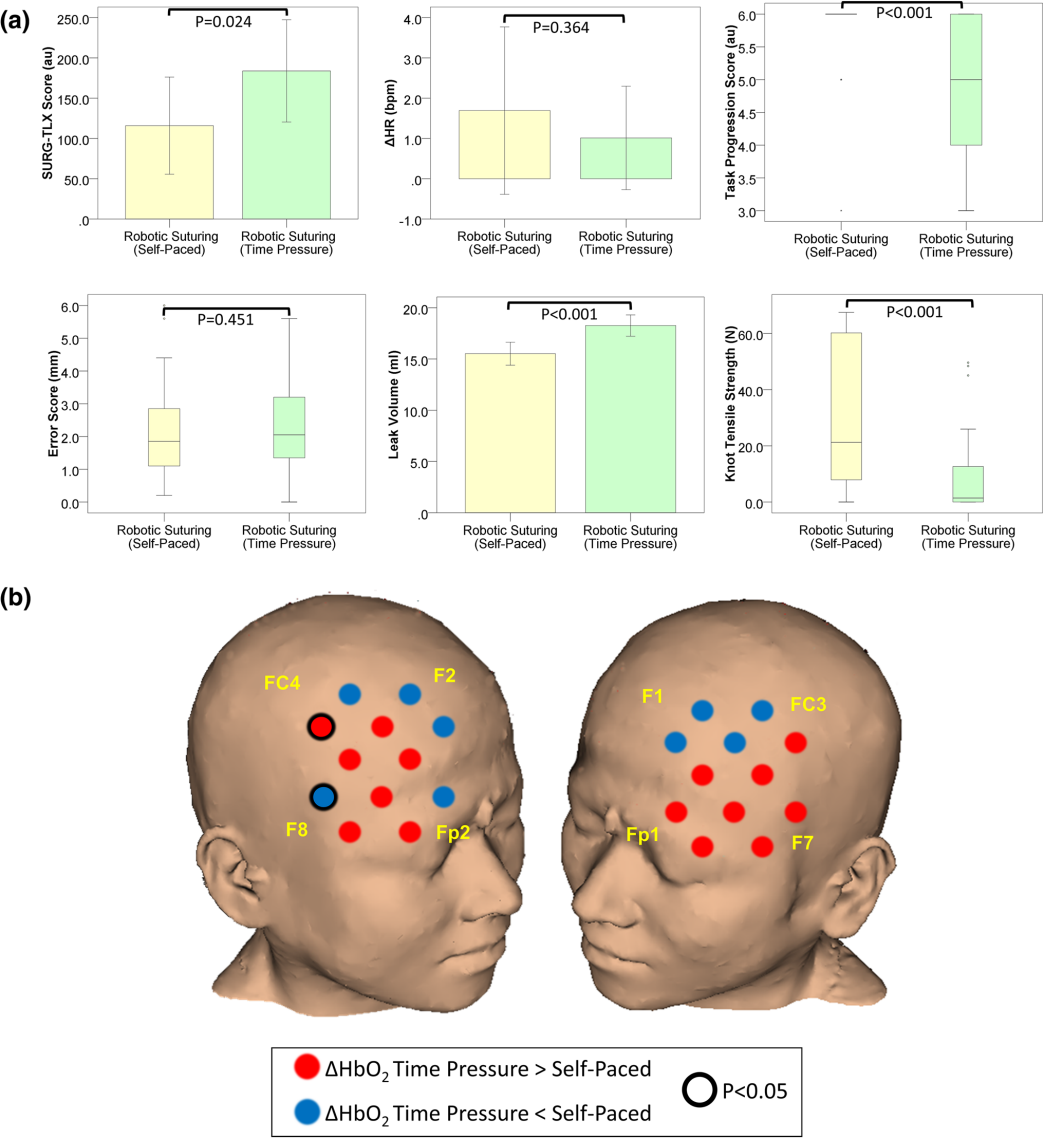
(**a**) Subjective workload (SURG-TLX) scores, heart rate, and technical performance in self-paced (yellow bars) and time pressure (green bars) conditions during robotic suturing. Error bars represent the 95% confidence interval; (**b**) Comparison of the HbO_2_ response (ΔHbO_2_) in time pressure and self-paced conditions during robotic suturing. Channels exhibiting a smaller HbO_2_ response in the time pressure compared to the self-paced condition are blue, whereas those exhibiting a smaller response in the self-paced condition are red. Channels in which there was a significant difference (*p* < 0.05, Wilcoxon Signed Ranks test) in the HbO_2_ response between conditions are outlined (black circle). Reference points of the 10–20 system of optode placement are shown in yellow (right PFC: Fp2 = source 6, F8 = source 7, F2 = source 9, FC4 = source 10; left PFC: Fp1 = source 2, F7 = source 1, F1 = source 5, FC3 = source 4). *Au* arbitrary units, *ΔHR* change in heart rate from rest to task, *bpm* beats per minute, *mm* millimetres, *mL* millilitres, *N* Newtons.

Subjective workload was significantly higher in the TP compared to SP condition (mean SURG-TLX ± SD: SP = 115.9 ± 72.1 vs. TP = 183.9 ± 75.9; *t*(7) = − 2.881, *p* = 0.024). As Fig. 3a highlights there was no significant difference in ΔHR between conditions (*p* = 0.364) (Table 1).

#### Technical Performance

Under TP, there was a significant deterioration in technical skills [task progression score (median (IQR): SP = 6.0 (0.0) vs. TP = 5.0 (2.0); *z* = -3.767, *p* < 0.001), leak volume (mean ± SD: SP = 15.5 ± 1.3 mL vs. TP = 18.3 ± 1.2 mL; *t*(7) = − 8.712, *p* < 0.001), and knot tensile strength (median (IQR): SP = 21.2 (52.9) N vs. TP = 1.4 (13.1) N; *z* = − 4.982, *p* < 0.001)]. As illustrated in Fig. 3a, no significant between-condition difference in error score was observed (*p* = 0.451) (Table 1).

#### Cortical Haemodynamic Response

In the SP condition, ten channels exhibited task-related HbO_2_ increases, primarily in the bilateral DLPFC, and eight channels demonstrated a non-significant decrease in HHb. No channels exhibited both an increase in HbO_2_ and decrease in HHb. In the TP condition, fifteen channels exhibited task-induced HbO_2_ increases across the bilateral VLPFC and right DLPFC, and seven channels demonstrated a decrease in HHb, one of which reached statistical significance. As in the SP condition, no channels exhibited an increase in HbO_2_ along with a decrease in HHb. Furthermore, contrary to the results observed during laparoscopy, ΔHbO_2_ was greater in TP compared to the SP condition in fifteen channels located in the bilateral VLPFC (Fig. 3b). Similarly, the magnitude of the ΔHHb response was greater in TP versus SP in eighteen channels.

### Laparoscopy vs. Robotic Surgery (Self-Paced)

#### Subjective Workload and Heart Rate

**Figure 4:**
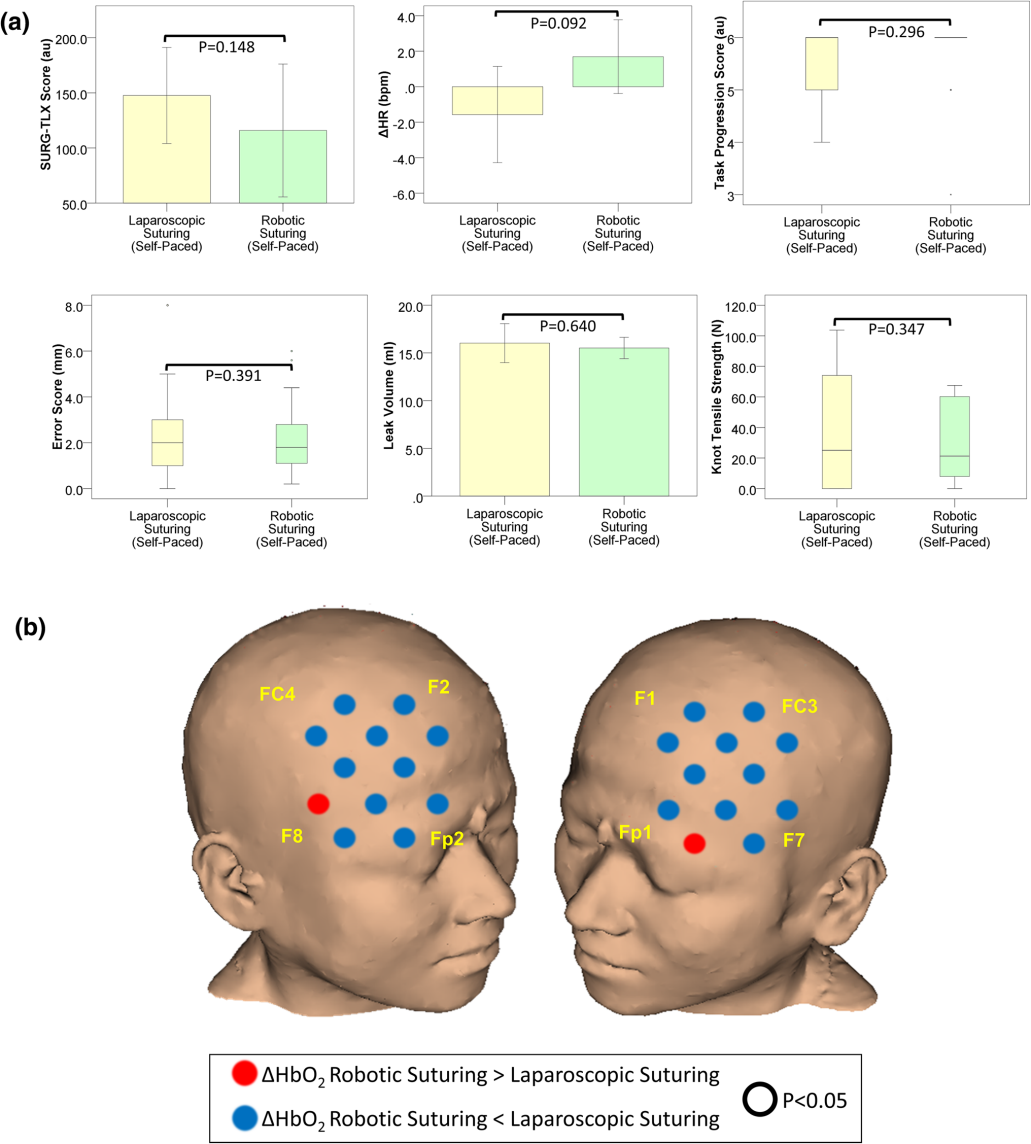
(**a**) Subjective workload (SURG-TLX) scores, heart rate, and technical performance in laparoscopic (yellow bars) and robotic (green bars) suturing in the self-paced condition. Error bars represent the 95% confidence interval; (**b**) Comparison of the HbO_2_ response (ΔHbO_2_) during laparoscopic and robotic suturing in the self-paced condition. Channels exhibiting a smaller HbO_2_ response during robotic suturing compared to laparoscopic suturing are blue, whereas those exhibiting a smaller response during laparoscopic suturing are red. Channels in which there was a significant difference (*p* < 0.05, Wilcoxon Signed Ranks test) in the HbO_2_ response between operative platforms are outlined (black circle). Reference points of the 10–20 system of optode placement are shown in yellow (right PFC: Fp2 = source 6, F8 = source 7, F2 = source 9, FC4 = source 10; left PFC: Fp1 = source 2, F7 = source 1, F1 = source 5, FC3 = source 4). *Au* arbitrary units, *ΔHR* change in heart rate from rest to task, *bpm* beats per minute, *mm* millimetres, *mL* millilitres, *N* Newtons.

SURG-TLX scores were lower during robotic suturing than laparoscopic suturing, but the difference was not statistically significant (*p* = 0.148). Similarly, as highlighted in Fig. 4a there was no significant difference in ΔHR between laparoscopic and robotic suturing (*p* = 0.092) (Table 1).

#### Technical Performance

There was no significant difference in task progression score (*p* = 0.296), error score (*p* = 0.391), leak volume (*p* = 0.640) or knot tensile strength (*p* = 0.347) between laparoscopic and robotic suturing in the SP condition (see Fig. 4a and Table 1).

#### Cortical Haemodynamic Response

In SP condition, ΔHbO_2_ was smaller during robotic suturing compared to laparoscopic suturing in 21 channels, however these differences did not reach significance (Fig. 4b).

### Laparoscopy vs. Robotic Surgery (Time Pressure)

#### Subjective Workload and Heart Rate

**Figure 5:**
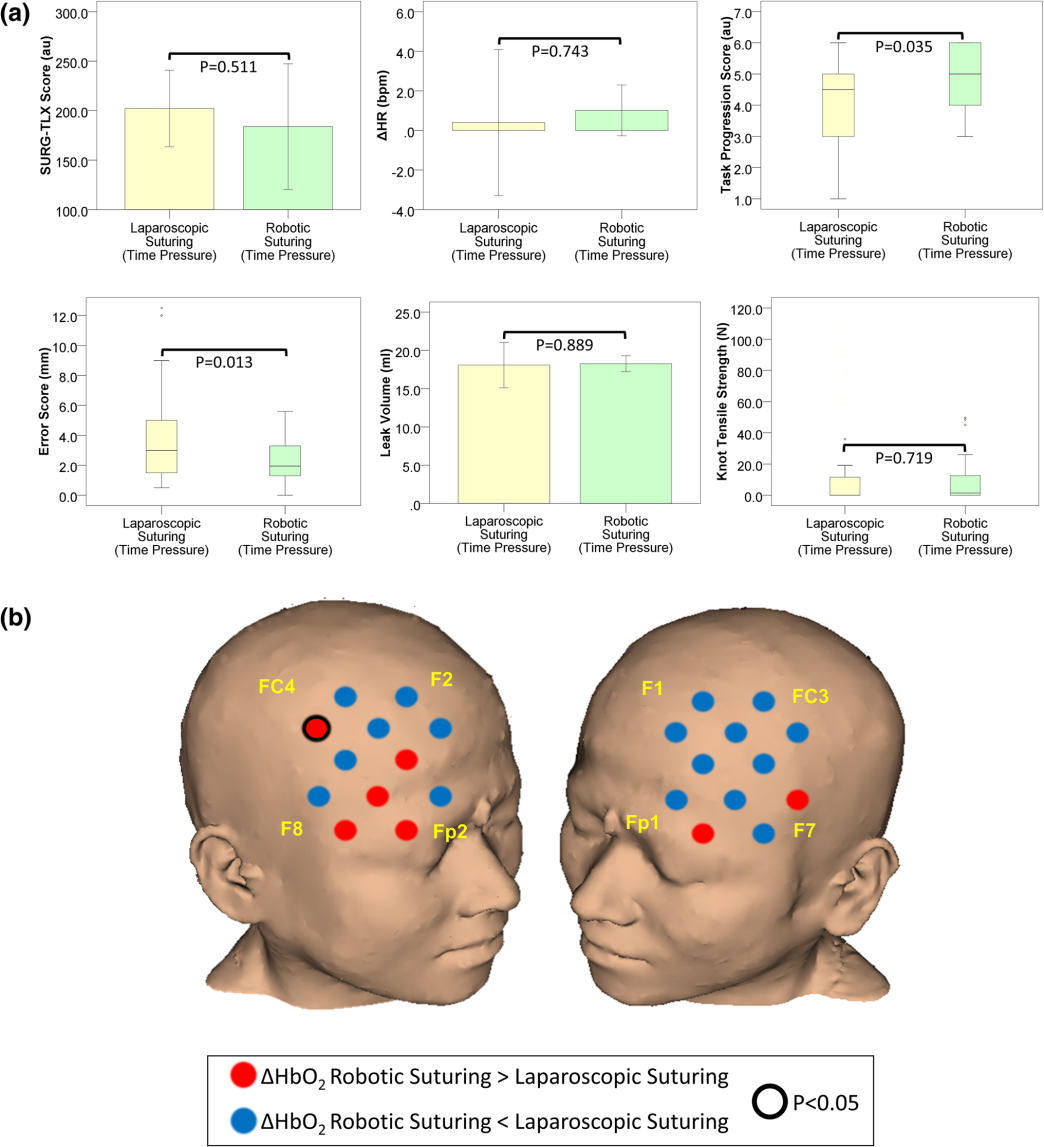
(**a**) Subjective workload (SURG-TLX) scores, heart rate, and technical performance in laparoscopic (yellow bars) and robotic (green bars) suturing in the time pressure condition. Error bars represent the 95% confidence interval; (**b**) Comparison of the HbO_2_ response (ΔHbO_2_) during laparoscopic and robotic suturing in the time pressure condition. Channels exhibiting a smaller HbO_2_ response during robotic suturing compared to laparoscopic suturing are blue, whereas those exhibiting a smaller response during laparoscopic suturing are red. Channels in which there was a significant difference (*p* < 0.05, Wilcoxon Signed Ranks test) in the HbO_2_ response between operative platforms are outlined (black circle). Reference points of the 10–20 system of optode placement are shown in yellow (right PFC: Fp2 = source 6, F8 = source 7, F2 = source 9, FC4 = source 10; left PFC: Fp1 = source 2, F7 = source 1, F1 = source 5, FC3 = source 4). *Au* arbitrary units, *ΔHR* change in heart rate from rest to task, *bpm* beats per minute, *mm* millimetres, *mL* millilitres, *N* Newtons.

SURG-TLX scores were lower during robotic suturing than laparoscopic suturing, but the difference did not reach significance threshold (*p* = 0.511). Similarly, as highlighted in Fig. 5a there was no significant difference in ΔHR between laparoscopic and robotic techniques (*p* = 0.743) (Table 1).

#### Technical Performance

The robotic technique resulted in significantly improved performance under TP with respect to task progression score (median (IQR): laparoscopic suturing = 4.5 (2.0) vs. robotic suturing = 5.0 (2.0); *z* = − 2.107, *p* = 0.035) and error score (median (IQR): laparoscopic suturing = 3.0 (3.6) mm vs. robotic suturing = 2.1 (1.9) mm; *z* = − 2.488, *p* = 0.013). There were no significant differences in leak volume (*p* = 0.889) or knot tensile strength (*p* = 0.719) between the robotic or laparoscopic platforms (see Fig. 5a and Table 1).

#### Cortical Haemodynamic Response

In the TP Condition, greater prefrontal ΔHbO_2_ was observed during robotic compared to laparoscopic suturing in seven channels, particularly those located in the right VLPFC (see Fig. 5b).

### Correlation Analysis

Across the all experimental conditions, only 4 out of 768 channels demonstrated statistically significantly (*p* < 0.05) correlations (*R* > 0.8) between heart rate and changes in HbO_2_. In the SP condition, there were no significant correlations between heart rate and changes in HbO_2_ in any channel in any subject during laparoscopic suturing, and in one channel (channel 19 in subject 2) during robotic suturing. In the TP condition, significant correlations were observed in two channels (channels 9 and 21 in subject 3) during laparoscopic suturing, and in one channel (channel 4 in subject 1) during robotic suturing (Supplementary Fig. 1).

## Discussion

The disparity in operator prefrontal cognitive demands between laparoscopy and robotic surgery was explored for a surgical suturing task and was further contrasted by varying temporal urgency. The results demonstrate the benefits of the robotic platform during episodes of high mental demand. In the comparatively calm conditions of the self-paced paradigm, there was no discernible difference in technical performance between the two operative platforms. However, when the same skill had to be recreated with greater sense of temporal urgency, such as would be required to suture a bleeding vessel, the robotic platform enabled improved accuracy, reduced technical errors and accelerated task progression. Critically, these performance improvements during the robotic task were coupled with interesting differences in the allocation of prefrontal resources compared to laparoscopy.

Both self-paced and time-pressure conditions were characterized by a general reduction in the magnitude of the prefrontal responses when operating with the robotic platform versus conventional laparoscopy. However, under temporal demand, the robotic platform was associated with greater responses across the right VLPFC, an area important for vigilance, resistance to environmental distraction, and attentional control,^21^,^27^ as well as inappropriate motor response suppression.^3^ This would suggest improvements in technical performance observed during robotic suturing under time pressure compared to laparoscopic suturing reflect more focused attention and greater task engagement that the robotic platform allows during stressful conditions, or alternatively that greater prefrontal resources are required to operate with the robotic platform with which trainees were less accustomed. In this regard, our previous work has shown that task novelty leads to a greater prefrontal response indicative of greater attentional demands^22^ which may explain the greater activation observed during robotic suturing. However, there was no significant difference in subjective workload (SURG-TLX) score between platforms which suggests that, although participants were less experienced with the robotic platform, they did not find it more demanding than the laparoscopic approach.

Laparoscopic surgery has been shown to be more mentally demanding and/or stressful compared with robotic surgery.^31^,^37^,^40^ The current study is the first to show that this disparity is related to diminished prefrontal engagement and attentional control during laparoscopy compared with the robotic approach. Whilst there have been no previous studies investigating this disparity from a neurocognitive perspective, literature from outside medicine have similarly demonstrated that mentally challenging tasks are associated with attenuated prefrontal responses.^1^,^13^,^16^,^26^,^34^ For example, in a fNIRS study by Lin *et al*.,^26^ subjects completed a walking task with or without a concurrent n-back working memory task.^26^ The most mentally demanding conditions (*i.e.,* dual task performance) resulted in slower gait speed, greater gait variability, and diminished prefrontal oxygenation.^26^ In another fNIRS study prefrontal activation was assessed during a complex naval air warfare management task, in which task load was manipulated by changing the number of aircrafts to be managed (6, 12, 18, or 24) and by introducing an auditory memory task.^16^ During the highest workload condition, mean cortical oxygenation decreased coupled with a deterioration in task performance.^16^ In line with findings from these fNIRS studies, Rowe *et al*.,^34^ used fMRI to scan subjects performing a finger movement task during which their attention was directed either towards their actions (low cognitive demand) or towards a visual search task (high cognitive demand).^34^ During attention-to-action, prefrontal activation increased compared with unattended performance of the same task.^34^ Finally, Al-Shargie *et al*.,^1^ utilised multimodal imaging (EEG and fNIRS) to measure prefrontal activity during a mental arithmetic task performed under time pressure and with negative feedback.^1^ The results showed that the stressful condition led to diminished activation in the ventrolateral PFC,^1^ in line with current study findings.

As important as the direct between-platform comparisons are the within-platform differences in workload, performance and brain responses associated with switching between self-paced and time pressure modes. Regardless of the operative platform, time pressure led to greater perceived workload and significant technical performance decline. Therefore, despite performance advantages of the robotic platform compared to laparoscopy when temporal demands are high, performance on the robot under pressure was substantially inferior to self-paced performance. Although time pressure-related deterioration in technical performance and escalation in subjective workload transcends operative platform, prefrontal responses associated with switching to a temporal demand were observed to be platform-dependent. Specifically, high temporal demand during laparoscopy was associated with a relative decrease in ΔHbO_2_. Similar prefrontal ‘deactivation’ responses during high workload states have also been observed during mental arithmetic,^1^ simulated piloting^13^ and military command tasks,^16^ and are thought to be due to disruption of executive control processes,^21^,^24^ distraction of participants with task-irrelevant cues,^29^ or neuro-hormonal inhibition of prefrontal synaptic activity.^2^ In contrast, robotic suturing under time pressure evoked a relative increase in prefrontal cortical responses across lateral PFC regions. This implies that whilst there may be a mismatch between workload and prefrontal haemodynamic responses during laparoscopic suturing under time pressure, there is improved alignment between escalating mental demands and prefrontal activation during robotic surgery.

Here, widespread ΔHbO_2_ increases under time pressure, suggest greater brain-resource allocation to deal with the increasing mental demands, an observation which is corroborated by others in the context of working memory tasks,^5^ air traffic control tasks,^6^ and warship command tasks.^16^ For example, Ayaz *et al*.,^6^ used fNIRS to assess prefrontal activation in air traffic controllers as they monitored an increasing number of aircrafts, and observed that oxygenation in the lateral PFC increased in-line with escalating mental demands.^6^ Despite the alignment between temporal pressure, subjective workload and prefrontal responses during robotic surgery it is interesting to note that PFC-related increase in attention and concentration did not manifest in performance stabilization. It mattered little whether PFC deactivations (laparoscopy) or enhanced PFC activations (robotic) were observed, high temporal demand still led to technical performance degradation. However, as described above, robotic performance was superior to laparoscopic under high temporal demands, possibly due to improved alignment between workload and prefrontal activation, and enhanced attentional control and task engagement. It should be noted, that despite significant improvement in task progression and error with robotic suturing compared to the laparoscopic approach under time constraints, there were no differences in leak volume or knot tensile strength. This may have been a result of experimental design. For example, subjects were not permitted to tie additional knots even if they felt the defect was not adequately closed, therefore any potential between-platform differences in leak volume would have been masked. Regarding knot tensile strength, the robotic platform lacks the force feedback that the laparoscopic technique confers. Therefore, during robotic suturing, subjects would not have experienced the same tactile perception of knot tightness as they would have during laparoscopic knot tying.

High workload states, such as temporal demands, could precipitate a stress-induced increase in heart rate, prompting spurious haemodynamic responses.^38^ Indeed, associations between heart rate and right PFC activity have been reported.^7^,^39^ However, mental arithmetic was used in previous studies to increase cognitive demands^7^,^39^ which may not extrapolate to complex surgical tasks. Moreover, there were no significant differences in ΔHR between conditions or between platforms in the current study, and significant per-subject correlations between heart rate and changes in HbO_2_ were not endemic, suggesting the results reflect genuine differences in cognitive processing. Interestingly, these correlations were subject-specific rather than related to expertise or platform specific.

This is not the first paper to describe to the relative merits of robotic surgery compared to laparoscopy. Studies have shown that, compared to the laparoscopic approach, robotic surgery improves manual dexterity,^10^ reduces technical errors,^31^,^37^ and shortens learning curves in novices.^10^,^37^ Furthermore, robotic surgery has been shown to reduce cognitive burden^31^,^37^,^40^ and dampen the physiological stress response.^31^,^40^ Whilst these studies all provide evidence to support the benefits of robotic platforms, the current is the first to demonstrate the relative benefits in terms of improved task-related attention and concentration using direct and objective assessment of surgeons’ brain function during episodes of high mental workload.

## Conclusion

During episodes of high mental workload evoked by temporal demands the robotic platform leads to improved technical performance and greater alignment between temporal demands, subjective workload and prefrontal activation as compared to laparoscopy. Further work should seek to develop biofeedback interventions to modulate neural activation during temporal stress and high workload states with the aim of improving attention and concentration, enhancing performance and improving patient safety.

## Limitations

Changes in respiration can lead to fluctuations in partial pressure of CO_2_ (pCO_2_), altering cerebral blood flow through changes in cerebral vascular tone.^17^,^43^ Although respiration was not measured, time-pressure may precipitate stress-related hyperventilation, decreases in pCO_2_ and cerebral vasoconstriction, dampening HbO_2_ concentration change. Furthermore, significant lateral PFC haemodynamic change, may be confounded by temporalis muscle activity.^35^ Future assessment of the cytochrome c oxidase response, a more brain-specific biomarker of cerebral metabolism, may overcome these limitations.^12^,^19^ A pre-hoc power calculation was not possible as data from pre-existing studies was inadequate for a sample size estimation,^5^,^6^,^8^,^13^,^16^ which others have similarly not attempted.^5^,^6^,^8^,^13^,^16^ Only eight subjects participated with others citing time required (approximately 3 h per subject) as prohibitive. This notwithstanding, numerous dependant variables were measured in each subject—(Hb species concentrations, heart rate, *etc*.) and hence the multidimensional dataset comprised thousands of data points which increased the validity of the statistical analysis. Whilst the controlled laboratory environment and use of a bench-top laparoscopic trainer may not mirror the operating room, between-condition differences in SURG-TLX scores imply the 2 min restriction adequately recreated a sense of urgency and increased mental effort. Moreover, the face validity of low-cost box trainers is proven.^9^,^25^,^33^,^36^

## Electronic supplementary material

Below is the link to the electronic supplementary material.

Supplementary material 1 (TIFF 5664 kb)

Supplementary material 2 (TIFF 5630 kb)

Supplementary material 3 (TIFF 5877 kb)

Supplementary material 4 (TIFF 5893 kb)

Supplementary material 5 (DOCX 10 kb)

## References

[1] Al-Shargie F, Kiguchi M, Badruddin N, Dass SC, Hani AF, Tang TB (2016). Mental stress assessment using simultaneous measurement of EEG and fNIRS. Biomed. Opt. Express.

[2] Arnsten AFT (2009). Stress signalling pathways that impair prefrontal cortex structure and function. Nat. Rev. Neurosci..

[3] Aron AR, Robbins TW, Poldrack RA (2004). Inhibition and the right inferior frontal cortex. Trends Cogn. Sci..

[4] Arora S, Sevdalis N, Nestel D, Woloshynowych M, Darzi A, Kneebone R (2010). The impact of stress on surgical performance: a systematic review of the literature. Surgery.

[5] Ayaz, H., M. Izzetoglu, S. Bunce, T. Heiman-Patterson and B. Onaral. Detecting cognitive activity related hemodynamic signal for brain computer interface using functional near infrared spectroscopy. In: 3rd International IEEE/EMBS Conference on Neural Engineering, 2007. pp. 342–345.

[6] Ayaz H, Shewokis PA, Bunce S, Izzetoglu K, Willems B, Onaral B (2012). Optical brain monitoring for operator training and mental workload assessment. Neuroimage.

[7] Brugnera A, Zarbo C, Adorni R, Tasca GA, Rabboni M, Bondi E, Compare A, Sakatani K (2017). Cortical and cardiovascular responses to acute stressors and their relations with psychological distress. Int. J. Psychophysiol..

[8] Bunce, S. C., K. Izzetoglu, H. Ayaz, P. Shewokis, M. Izzetoglu, K. Pourrezaei and B. Onaral. Implementation of fNIRS for monitoring levels of expertise and mental workload. International Conference on Foundations of Augmented Cognition. Orlando, Florida USA: Springer Berlin Heidelberg, 2011. pp. 13–22.

[9] Campo R, Reising C, Van Belle Y, Nassif J, O’Donovan P, Molinas CR (2010). A valid model for testing and training laparoscopic psychomotor skills. Gynecol. Surg..

[10] Chandra V, Nehra D, Parent R, Woo R, Reyes R, Hernandez-Boussard T, Dutta S (2010). A comparison of laparoscopic and robotic assisted suturing performance by experts and novices. Surgery.

[11] Cope M, Delpy DT, Reynolds EO, Wray S, Wyatt J, van der Zee P (1988). Methods of quantitating cerebral near infrared spectroscopy data. Adv. Exp. Med. Biol..

[12] de Roever I, Bale G, Cooper RJ, Tachtsidis I (2017). Functional NIRS measurement of cytochrome-C-oxidase demonstrates a more brain-specific marker of frontal lobe activation compared to the haemoglobins. Adv. Exp. Med. Biol..

[13] Durantin G, Gagnon JF, Tremblay S, Dehais F (2014). Using near infrared spectroscopy and heart rate variability to detect mental overload. Behav. Brain Res..

[14] Hubert N, Gilles M, Desbrosses K, Meyer JP, Felblinger J, Hubert J (2013). Ergonomic assessment of the surgeon’s physical workload during standard and robotic assisted laparoscopic procedures. Int. J. Med. Robot.

[15] Huppert TJ, Diamond SG, Franceschini MA, Boas DA (2009). HomER: a review of time-series analysis methods for near-infrared spectroscopy of the brain. Appl. Opt..

[16] Izzetoglu K, Bunce S, Onaral B, Pourrezaei K, Chance B (2004). functional optical brain imaging using near-infrared during cognitive tasks. Int. J. Human Comput. Interact..

[17] Jordan J, Shannon JR, Diedrich A, Black B, Costa F, Robertson D, Biaggioni I (2000). Interaction of carbon dioxide and sympathetic nervous system activity in the regulation of cerebral perfusion in humans. Hypertension.

[18] Jurcak V, Tsuzuki D, Dan I (2007). 10/20, 10/10, and 10/5 systems revisited: their validity as relative head-surface-based positioning systems. Neuroimage.

[19] Kolyva C, Ghosh A, Tachtsidis I, Highton D, Cooper CE, Smith M, Elwell CE (2014). Cytochrome c oxidase response to changes in cerebral oxygen delivery in the adult brain shows higher brain-specificity than haemoglobin. Neuroimage.

[20] Lanfranco AR, Castellanos AE, Desai JP, Meyers WC (2004). Robotic surgery: a current perspective. Ann. Surg..

[21] Langner R, Eickhoff SB (2013). Sustaining attention to simple tasks: A meta-analytic review of the neural mechanisms of vigilant attention. Psychol. Bull..

[22] Leff DR, Elwell CE, Orihuela-Espina F, Atallah L, Delpy DT, Darzi AW, Yang GZ (2008). Changes in prefrontal cortical behaviour depend upon familiarity on a bimanual co-ordination task: an fNIRS study. Neuroimage.

[23] Leff DR, Orihuela-Espina F, Athanasiou T, Karimyan V, Elwell C, Wong J, Yang GZ, Darzi AW (2010). “Circadian cortical compensation”: a longitudinal study of brain function during technical and cognitive skills in acutely sleep-deprived surgical residents. Ann. Surg..

[24] Leff DR, Yongue G, Vlaev I, Orihuela-Espina F, James D, Taylor MJ, Athanasiou T, Dolan R, Yang GZ, Darzi A (2017). “Contemplating the Next Maneuver”: functional neuroimaging reveals intraoperative decision-making strategies. Ann. Surg..

[25] Li MM, George J (2017). A systematic review of low-cost laparoscopic simulators. Surg. Endosc..

[26] Lin M-IB, Lin K-H (2016). Walking while performing working memory tasks changes the prefrontal cortex hemodynamic activations and gait kinematics. Front. Behav. Neurosci..

[27] Love T, Haist F, Nicol J, Swinney D (2006). A functional neuroimaging investigation of the roles of structural complexity and task-demand during auditory sentence processing. Cortex.

[28] Miller EK, Cohen JD (2001). An integrative theory of prefrontal cortex function. Annu. Rev. Neurosci..

[29] Modi HN, Singh H, Orihuela-Espina F, Athanasiou T, Fiorentino F, Yang GZ, Darzi A, Leff DR (2017). Temporal stress in the operating room: brain engagement promotes “Coping” and disengagement prompts “Choking”. Ann. Surg..

[30] Modi HN, Singh H, Yang GZ, Darzi A, Leff DR (2017). A decade of imaging surgeons’ brain function (part II): a systematic review of applications for technical and nontechnical skills assessment. Surgery.

[31] Moore LJ, Wilson MR, McGrath JS, Waine E, Masters RSW, Vine SJ (2015). Surgeons’ display reduced mental effort and workload while performing robotically assisted surgical tasks, when compared to conventional laparoscopy. Surg. Endosc..

[32] Orihuela-Espina F, Leff DR, James DRC, Darzi AW, Yang GZ (2018). Imperial College near infrared spectroscopy neuroimaging analysis framework. Neurophotonics.

[33] Palter VN, Orzech N, Aggarwal R, Okrainec A, Grantcharov TP (2010). Resident perceptions of advanced laparoscopic skills training. Surg. Endosc..

[34] Rowe J, Friston K, Frackowiak R, Passingham R (2002). Attention to action: specific modulation of corticocortical interactions in humans. Neuroimage.

[35] Schecklmann M, Mann A, Langguth B, Ehlis AC, Fallgatter AJ, Haeussinger FB (2017). The temporal muscle of the head can cause artifacts in optical imaging studies with functional near-infrared spectroscopy. Front. Hum. Neurosci..

[36] Schreuder HW, van den Berg CB, Hazebroek EJ, Verheijen RH, Schijven MP (2011). Laparoscopic skills training using inexpensive box trainers: which exercises to choose when constructing a validated training course. BJOG.

[37] Stefanidis D, Wang F, Korndorffer JR, Dunne JB, Scott DJ (2010). Robotic assistance improves intracorporeal suturing performance and safety in the operating room while decreasing operator workload. Surg. Endosc..

[38] Tachtsidis I, Scholkmann F (2016). False positives and false negatives in functional near-infrared spectroscopy: issues, challenges, and the way forward. Neurophotonics.

[39] Tanida M, Sakatani K, Takano R, Tagai K (2004). Relation between asymmetry of prefrontal cortex activities and the autonomic nervous system during a mental arithmetic task: near infrared spectroscopy study. Neurosci. Lett..

[40] van der Schatte Olivier RH, van‘t Hullenaar CDP, Ruurda JP, Broeders IAMJ (2008). Ergonomics, user comfort, and performance in standard and robot-assisted laparoscopic surgery. Surg. Endosc..

[41] Wilson MR, Poolton JM, Malhotra N, Ngo K, Bright E, Masters RS (2011). Development and validation of a surgical workload measure: the surgery task load index (SURG-TLX). World J. Surg..

[42] Yang G-Z, Cambias J, Cleary K, Daimler E, Drake J, Dupont PE, Hata N, Kazanzides P, Martel S, Patel RV, Santos VJ, Taylor RH (2017). Medical robotics—Regulatory, ethical, and legal considerations for increasing levels of autonomy. Sci. Robot..

[43] Yoon S, Zuccarello M, Rapoport RM (2012). pCO(2) and pH regulation of cerebral blood flow. Front. Physiol..

