# Supplementary material for: Robotic Surgery Improves Technical Performance and Enhances Prefrontal Activation During High Temporal Demand
Source: Ann Biomed Eng. 2018 Jun 4;46(10):1621–36. doi: 10.1007/s10439-018-2049-z (PMC6153983; doi:10.1007/s10439-018-2049-z)
Supplement: Supplementary file 5 — Supplementary material 5 (DOCX 10 kb) [file 10439_2018_2049_MOESM5_ESM.docx]

**Supplementary Figure 1.** Correlation maps demonstrating channel-wise correlations between heart rate and changes in oxygenated haemoglobin concentration per subject during (a) self-paced laparoscopic suturing, (b) self-paced robotic suturing, (c) time pressured laparoscopic suturing, and (d) time pressured robotic suturing. Channels in which there are significantly (p<0.05) strong correlations (R>0.8) are shown as white.
